# Supplementary material for: Chromothripsis during telomere crisis is independent of NHEJ, and consistent with a replicative origin
Source: Genome Res. 2019 May;29(5):737–49. doi: 10.1101/gr.240705.118 (PMC6499312; doi:10.1101/gr.240705.118)
Supplement: Supplemental Material [file supp_gr.240705.118_Supplemental_file_1.zip › contigs/annotated_contigs/DB48/contig.4.DB48_length_592_mean_cov_9.03547297297.docx]

**DB48_length_592_mean_cov_9.03547297297**

```
ATTCGCCTGCCTTGGCCTCCCAAAGTGCTGGGATTACAGGCATGAGCCACTGTGCCCGG|G|CTGTGTTGATTTATTTTATTTTATTTT
 >chr12:65136038-65136097 + E=9e-18                          >chr16:26753405-26753438 -
ATTTTA|T|TTTATTTTATTTTATTTTATTTTATTTTATTTTATTTTATTTTATTTTATTTTTATGTGACGGAGT|ATCCCTGGAGTGC
 E=4e-08 p=0e+00  >chr9:76965762-76965828 + E=5e-22 p=0e+00
AGTGGCACGATCTCAGCTCACTGCAACCTCTGCCTTCTGGGATCAAGCAATTCTCCT|GCCTCAGCCTCCTGAGTAGCTGGGATTACAG
                                                          >chr1:29000201-29000564 - E=6e
GCGCCCGCCACCATGCCTGGCTAATTTTTGTATTTTTAGTAGAGACAGGGTTTCATCATGTTGGCCAGGCTGGTCTTGAACTCCTGACC
-196
TCGTGATCCACCCACCACGGCCTCCCAAAGTGCTGGGACAGGCATGAGACACCACGCCTGGCCTAATTTCTTTACTTCTTTGTAGACAT

GAGGTCTTGCTATGTTGCCTAGCCTGGTGTCAAACTCCTGGGCTCAAGCAATCCTCTCACCTCGGCCTCCCAAAGTGCTGAGATTAAAG

ACCTGAGCGACTGTGCCCAGCCTGTAGTTATTTCCAAAGACAAAAACAACACTTTGAAACTAAG
```
